# Greener Nanocomposite Polyurethane Foam Based on Sustainable Polyol and Natural Fillers: Investigation of Chemico-Physical and Mechanical Properties

**DOI:** 10.3390/ma13010211

**Published:** 2020-01-04

**Authors:** Ferdinando De Luca Bossa, Chiara Santillo, Letizia Verdolotti, Pietro Campaner, Andrea Minigher, Laura Boggioni, Simona Losio, Francesca Coccia, Salvatore Iannace, Giuseppe C. Lama

**Affiliations:** 1 Institute for Polymers, Composites and Biomaterials-CNR, 80055 Portici (NA), Italy; fe.delucabossa@gmail.com (F.D.L.B.); chiara.santillo@unina.it (C.S.); salvatore.iannace@cnr.it (S.I.); giuseppe.lama@ipcb.cnr.it (G.C.L.); 2 AEP Polymers Srl, 34149 Basovizza, Trieste, Italy; pietro.campaner@aeppolymers.com (P.C.); andreaminigher@aeppolymers.com (A.M.); 3 Institute for Chemical Science and Technologies -CNR, 20133 Milano, Italy; laura.boggioni@cnr.it (L.B.); simona.losio@cnr.it (S.L.); francescacoccia84@gmail.com (F.C.)

**Keywords:** nanocomposite foams, milled walnut shell, milled cellulose, diatomite, sustainable polyurethane

## Abstract

Nowadays, the chemical industry is looking for sustainable chemicals to synthesize nanocomposite bio-based polyurethane foams, PUs, with the aim to replace the conventional petrochemical precursors. Some possibilities to increase the environmental sustainability in the synthesis of nanocomposite PUs include the use of chemicals and additives derived from renewable sources (such as vegetable oils or biomass wastes), which comprise increasingly wider base raw materials. Generally, sustainable PUs exhibit chemico-physical, mechanical and functional properties, which are not comparable with those of PUs produced from petrochemical precursors. In order to enhance the performances, as well as the bio-based aspect, the addition in the polyurethane formulation of renewable or natural fillers can be considered. Among these, walnut shells and cellulose are very popular wood-based waste, and due to their chemical composition, carbohydrate, protein and/or fatty acid, can be used as reactive fillers in the synthesis of Pus. Diatomite, as a natural inorganic nanoporous filler, can also be evaluated to improve mechanical and thermal insulation properties of rigid PUs. In this respect, sustainable nanocomposite rigid PU foams are synthesized by using a cardanol-based Mannich polyol, MDI (Methylene diphenyl isocyanate) as an isocyanate source, catalysts and surfactant to regulate the polymerization and blowing reactions, H_2_O as a sustainable blowing agent and a suitable amount (5 wt%) of ultramilled walnut shell, cellulose and diatomite as filler. The effect of these fillers on the chemico-physical, morphological, mechanical and functional performances on PU foams has been analyzed.

## 1. Introduction

Since the publication of the principles of “green chemistry” at the end of 1990s by Anastas and Werner [1], a great effort related to improving the treatment, synthesis and disposal of chemical substances was made by scientific community. Attention has principally gone to those issues aimed to protect the environment and preserve its resources for future generations. For these reasons, given that the crude oil is a limited resource, research has to develop other processes to produce materials starting from eco-friendly and sustainable source [2,3,4,5]. In this contest, biomass, waste and by-product from different industrial sectors have gained interest as sustainable source to obtain chemicals that can be used (or re-used) in several industries [6]. Vegetable oil [2] and carbohydrates are another interesting type of biomass as starting compounds in many industrial sectors such as bio-refinery and polymer industry [7].

The same drawbacks have motivated continued research focus in developing green routes by using sustainable or renewable sources also to develop bio-based Polyurethanes [8,9].

Polyurethanes (PUs) are one of the most versatile classes of polymers. Their morphology, as well as their chemical structure, can be tailored to fit specific requirements by a correct selection of the precursors. Thanks to their versatility, PUs are one of the most requested materials, used in several sectors of the industry, such as thermo-insulator in the building, automotive, furniture, bedding, refrigerators, wood substitutes and packaging fields [10,11]. For their preparation, a reaction between an oligomeric polyol and a diisocyanate (or polyisocyanate) mainly derived from treatment of crude oil is required [12]. Furthermore, the PUs can be used as a matrix to prepare high performance composite or nanocomposite foam materials to be applied as advanced materials in specific fields. The introduction of micro or nano-particles [11,12] within a polyurethane foam matrix permit to overcome some limitation of conventional PUs. For instance, PU foam composites highlight properties such as abrasion resistance, fire resistance, high flexibility, high durability, anti-aging, high impact strength, good thermal stability at high and low temperature, which permit them to also be applied in sensible sectors such as aerospace and more.

Many studies have demonstrated that several directions can be taken to make an eco-friendlier production process of PUs, and all of them are strongly encouraged.

In this respect, in the synthesis of PU foams, two sustainable directions can be considered: the total replacement of synthetic polyols with green polyols [13,14] or the elimination of isocyanatic source (non-isocyanate polyurethane, NIPU).

Generally, the preparation of NIPU [15] occurs through a reaction (i.e., an environmental friendly polyaddition process or un-sustainable polycondensation or ring opening polymerization) of a polyamine with a cyclic carbonate, in which typically the amine comes from a petrochemical source and cyclic carbonate from sustainable sources (such as biomass, soybean and rapeseed oils, etc.) [9,16].

In the case of PUs with green polyols, the PUs is obtained by polyaddition reaction of bio-based polyol and conventional isocyanate. One of the most investigated routes uses “bio-based polyols” [8,10] derived from agricultural (biomass) and industrial waste stream [3,4,5] to produce PUs. In this respect, among several renewable resources, Suresh et al. [13] used cashew nut shell liquid (CNSL), derived through cashew processing. Thanks to its peculiar chemical structure (alkyl phenolic moiety with an un-saturated C15 side chain) [13,14]), this compound resulted to be an optimal candidate to synthesize PU foams with enhanced mechanical and functional properties [13,14].

As described before, in both cases, the produced PUs are partially renewable, because for PUs with bio-based polyols there is the isocyanate (synthetic precursor) and in the case of NIPU there are organic polyamines (synthetic precursor) [9,15,16].

Either way, the produced polyurethane foams have to achieve performances (in terms of mechanical, thermal degradation, fire resistance as well as functional properties) at least equal to the fossil-based reference material, must have a consolidate technology of production and must comply with regulations of “conventional PU foams”. This, along with the cost, could nowadays be a limiting hurdle for commercial-scale production [17]. In this instance, the research still has a great task ahead of it, before these challenges become reality. In this work, sustainable polyurethane composite foams, starting from a Mannich-based polyol, produced by CNSL [13,14], and three natural fillers, such as walnut shells, cellulose and diatomite, were synthesized. Walnut shells are agricultural or agro-food residues, due to the high amount of walnut production in the world (around 3.7 million tons [18]); walnut shells result in serious environmental problems in several countries. Cellulose is a natural material obtained by several sustainable or renewable sources such as biomass [19] or industrial paper. Diatomite is a sedimentary amorphous silica rock, frustules [20], that possesses useful properties in a wide range of application fields. Their peculiarities are related to the presence of a fine-porous structure (at nano-size scale) [20]) and high surface area on one hand and lightness and low thermal conductivity on the other.

These fillers were selected for their availability (wide natural availability), their chemical composition (-OH functional groups) and structure, which interact with the polyurethane matrix inducing an improvement of mechanical or functional performances [10,21]. The effect of those fillers on the thermal, mechanical and chemical properties of the produced composite foams were investigated and compared to the pristine polyurethane foam.

## 2. Materials and Methods

### 2.1. Chemicals

MDI (Methylene diphenyl isocyanate) (SUPRASEC 2085 with NCO content equal to 30.5 wt%) and sustainable Mannich polyol (code GX9104 with OH index equal to 245 mg KOH/g) were kindly provided by Huntsman srl (Ternate, Italy) and Cardolite Corporation (Bristol, PA, USA), respectively. CH_3_COOK and Niax PM40 additives were used to control both polymerization and blowing reactions, and L6164 was used as surfactant to stabilize the cells in the PU foam; both were kindly provided by Momentive (Terni, Italy). Distilled H_2_O was used as blowing agent. Cellulose powder (labeled C) by Sigma Aldrich (Milano, Italy, Code-C8002) and walnut shell (labeled WS, food waste) were milled (for 120 min) by using a pestle mill equipment until obtaining a granulometry around 8 and 8.5 µm, respectively. Diatomite (labeled D, Celite code-545C) flux calcined diatomaceous earth filter aid, was supplied by Imerys (Murat, France); the average structural features of frustules are diameter of ~5 µm, length of 10−20 µm, regularly spaced rows of pores with diameter of ~400−500 nm located along the frustule walls, see the micrographs in Figure 4b).

All the materials were used as received.

### 2.2. Foams Nanocomposite Preparations, PUs

PUs were synthesized by setting the NCO/OH ratio equal to 1.4. Different foams samples were obtained by adding a suitable amount of catalysts, surfactant, H_2_O and dried powder (walnut shell, cellulose or diatomite) to the polyol. After mixing, through a magnetic stirring plate, a definite amount of MDI was added to the aforementioned mixture and mixed. The resulting composite mixture was left to rise in a closed rectangular mold (10 × 20 × 5 cm^3^), and the produced foams were subsequently cured at 40 °C for 2 h before any characterization. A reference PU formulation was also prepared for proper comparison. In Table 1, the formulations, along with their sample codes, are reported. In order to minimize boundary effects and assure that the cellular structure is homogenous, chemical-physical and mechanical characterizations have been performed on samples cut from the center of the plates.

## 3. Characterizations

### 3.1. Wide and Small Angle X-ray Scattering (WAXS/SAXS)

Wide (WAXS) and Small Angle X-ray Scattering (SAXS) were performed using an SAXSess camera (Anton Paar, Ashland, VA, USA) equipped with a two-dimensional (2D) imaging plate detector. 1.5418 Å wavelengths CuKα X-Rays were generated by a Philips PW3830 sealed tube generator source (40 kV, 50 mA) and slit collimated. Spectra of pristine polyurethane (PUG) and composite polyurethane foams (PUG-C, PUG-WS, PUG-D) were collected for 60 min. The dark current and background were subtracted from all scattering data [12].

### 3.2. Infrared Spectroscopy (FTIR)

FTIR spectra (Fourier-transform infrared spectroscopy) were recorded at room temperature by using a FT-IR spectrometer (model Frontier Dual Ranger, PerkinElmer, CT, USA) in attenuated total reflectance (ATR) mode from 400–4000 cm^−1^. ATR spectra were collected on the surface of the cubic foam sample before and after UV treatment. Spectra were recorded at 4 cm^−1^ resolution, and the reported results are the average of 64 scans [10,12,22].

The spectral region between 1400 and 1800 cm^−1^ was investigated, all the spectra were background-corrected [10,12] and subsequently were deconvoluted with OriginPro 8.0 software by using Gauss function.

### 3.3. Polarized Optical Microscopy (POM)

Optical microphotographs of the samples were recorded at room temperature with Polarized optical microscopy (POM) images, using an Olympus BX51 microscope. The PUG, PUG-C, PUG-WS and PUG-D samples were mildly grinded before POM analysis. The size of the spherulites have been determined by statistical analysis on a set of POM images by using ImageJ program.

### 3.4. Scanning Electron Microscopy (SEM)

Foam morphology was analyzed with a scanning electron microscope (model S8000, Tescan, Czech Republic). Small specimens were cut (0.5 × 0.5 × 0.5 cm^3^) from the middle of the foam in the direction of growth. The pieces were coated with gold with a sputter coater (Emscope SC500, London, UK) before observation.

### 3.5. Thermal Properties (Thermo-Gravimetric Analysis-TGA, Differential Scanning Calorimetric-DSC and Thermal Conductivity)

Thermal degradation behavior was evaluated by thermo-gravimetric analysis (TGA) that was carried out in nitrogen flow (flow rate = 40 mL/min) using a TGAQ500-TA Instruments (New Castle, DE, USA), at a heating rate equal to 10 °C/min in the temperature range from 30 °C to 1000 °C. Samples mass was approximately 10 mg.

The differential scanning calorimetric (DSC) analyses were conducted on a Q 1000, TA Instruments, (New Castle, DE, USA). Samples were first heated from −80 °C to 200 °C, and then subjected to cool up to −80 °C, and finally reheated to 200 °C. The rates of heating and cooling were 10 °C/min.

Thermal conductivity, λ, was evaluated by C-Therm Technologies (TCi) Analyzer.

### 3.6. Mechanical Properties

Mechanical compressive strength of composite foams was assessed according to ASTMD1621 by applying a compression load to specimens having a square section (4 × 4 × 4 cm^3^). The compression tests were carried out at room temperature and humidity, by using a universal testing machine (model CMT4304 from Shenzhen SANS Testing Machine Co. China, now MTS-USA) equipped with a 30 kN load cell and the cross-head speed was equal to 4 mm/min.

Foam density was calculated as the ratio between the weight and geometric volume of composite foams. The weight was measured by an analytical balance (model AB265-S from Mettler Toledo, Columbus, OH, USA) and sample dimensions were evaluated by a high-resolution caliper (model 500-181-30 from Mitutoyo, Kawasaki, Japan).

Five samples for each composition were analyzed and the average values and standard deviations were evaluated.

## 4. Results and Discussion

### 4.1. Wide and Small Angle X-ray Scattering (WAXS/SAXS) and Optical Polarized Microscopy (POM)

**Figure 1:**
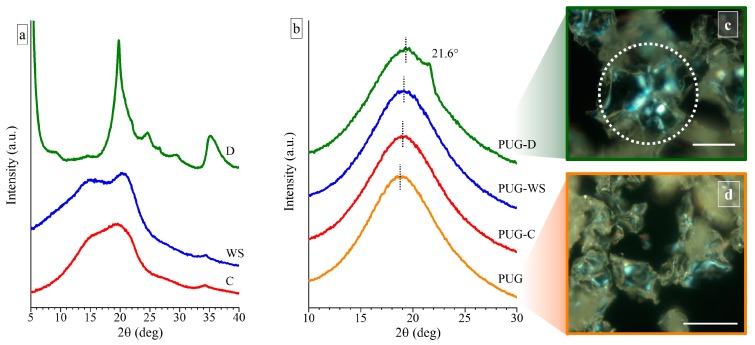
Wide Angle X-ray scattering (WAXS) profiles of (**a**) the milled cellulose (C), milled walnut shell (WS), and diatomite (D) fillers and of (**b**) the polyurethane composite foams PUG, PUG-C, PUG-WS and PUG-D. Polarized optical microscopy (POM) images of (**c**) the PUG-D and of (**d**) the pristine PUG. The scale bar in the POM images is 50 µm.

WAXS spectra of the milled cellulose (C), the milled walnut shell (WS) and of the diatomite (D), used as fillers in the preparation of polyurethane composite foams, are shown in Figure 1a.

The WAXS profile of the C shows the presence of two very broad halos (which are almost collapsed in only one broad halo) at 2θ values of ~19.57° and 15.28°, suggesting that the used C is characterized by a high degree of amorphous phase and only few and small crystals are present in a mixture of the Form I and Form II [19]. Two better-resolved diffraction peaks at 2θ values of 20.45° and 15.05° are observed in the WAXS spectrum of powders of the WS (Figure 1a), revealing the existence of crystals in the Form I and II of the cellulose and a higher crystallinity than the C.

For the diatomite (D), all diffraction peaks detected in the corresponding WAXS profile can be assigned by the attendance of different polymorphic forms of silica [12,23].

In order to study the influence of the different natural fillers on the crystallization process of the polyurethane foam, WAXS measurement were carried out on pristine PUG and on composite systems PUG-C, PUG-WS and PUG-D (Figure 1b).

The diffraction profiles of the pristine PUG and of the composite foams PUG-C and PUG-WS are characterized only by a broad halo centered at 2θ ≈ 19°, indicating that the systems are amorphous. The WAXS profile of the sample PUG-D shows a shoulder at 2θ = 21.6°, which is related to the appearance of a low crystallinity of the polyurethane (PU) [24]. In fact, since the maximum diffraction peak of the diatomite is located at 2θ = 19.8° (Figure 1b), the shoulder in the WAXS profile of the PUG-D can be attributed only to the crystallinity of the polymer. This result is also in agreement with the presence of spherulites, with a size of about 20–30 µm, in the POM image of the sample PUG-D (Figure 1c). Similar spherulitic structures were not found in the POM images of the pristine PUG (Figure 1d) and of the composite foams PUG-C and PUG-WS (data not shown). Probably, PUG, PUG-C, and PUG-WS systems that appear amorphous from WAXS analysis, are characterized by very small crystalline entities, which are difficult to detect by POM observations. Therefore, WAXS and POM data of the foams suggest that the different fillers participate in different manners on the crystallization process of the polyurethane and in particular, the diatomite induces an increase of the crystallinity acting as a nucleating agent on the crystallization of the polymer.

Furthermore, in the WAXS profiles of the PUG-C, PUG-WS and PUG-D, a shift is observed at slightly higher 2θ values of the amorphous halo compared to the 2θ value of the pristine PUG (from 2θ = 18.76° for the PUG to 19.02°, 19.09° and 19.35° for the PUG-C, PUG-WS and PUG-D, respectively). This effect is ascribed to a modification of the local microstructure of the polyurethane network, which likely arises from the ability of cellulose, walnut shell and diatomite to become involved in the building up of the organic network through crosslinking reactions among -OH groups of the fillers and -NCO groups of the isocyanate [25].

The polyurethane composite foams were further investigated by SAXS experiments in order to understand how the interaction of the different fillers with the PU network intervenes on the PU morphology, and finally, to correlate the PU morphology to the final properties (such as thermal and mechanical properties) of the systems.

SAXS analysis has been widely used for studying PU morphology [26,27,28,29,30] because these materials are usually random segmented copolymers of alternating flexible soft-segments (SS) and more rigid hard-segments (HS) containing urethane and/or urea domains. Hence, PUs typically show a microphases-separated morphology where the different electronic densities among SS and HS is responsible of the broad knee scattering feature present in their SAXS patterns. Thus, the interdomain distance between HS (*L*) can be estimated by using the Bragg’s law:

*L* = 2*π*/*q*_max_ (1)

where *q*_max_ is the maximum of the intensity of the scattering vector (*q*), determined from the Lorentz corrected plot (*I*(*q*)·*q*^2^ − *q*). Moreover, the electron densities difference can be quantitatively evaluated by the invariant of scattering *Q* as follows:

(2) $$Q=\int_{0}^{\infty }q^{2}I\left(q\right)dq$$

The value of *Q* describes the electron density fluctuation of polymer and is a good approximation to estimate the overall degree of phase separation (DPS) in PU [24,29].

**Figure 2:**
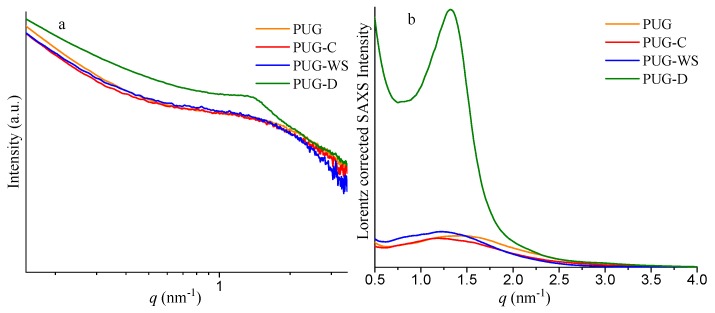
Small angle X-ray scattering (SAXS) profiles (**a**) and corresponding Lorentz corrected plots (**b**) of the polyurethane composite foams PUG, PUG-C, PUG-WS and PUG-D.

SAXS profiles of the polyurethane composite foams and the corresponding Lorentz corrected plots are reported in Figure 2, while the values of *q*_max_, *L* and *Q* are listed in Table 2.

The samples PUG-C and PUG-WS show values of *L* of 5.32 nm and 5.15 nm, respectively, which are higher than the *L* value of 4.42 nm of the pristine PUG. Usually, an increase of the interdomain distance between HS is also related to an increase of the size of the domains and to an increase of the *Q* value. As reported in Table 2, the *Q* values for the PUG-C and PUG-WS are slightly lower than the *Q* value found for the PUG. These results can be reasonable with the formation of some urea and/or urethane hard domains in the PUG-C and PUG-WS, which are not incorporated with the polymer network [28], causing a disruption of the long range connectivity between the HS. These unattached urea/urethane-HS do not contribute to the phase separation degree in the polyurethane, because they are isolated domains and are more mobile than those within the PU microdomains, justifying the slightly decrease of the *Q* values and the increment of the *L* values of polyurethane composite foams containing C and WS as fillers.

SAXS analysis of the PUG-D system (Figure 2 and Table 2) demonstrates that the diatomite plays a completely different role from those observed for cellulose and walnut shell in the morphology of the PU. In fact, the presence of the D induces an increase of the interdomain distance (from 4.42 nm for the PUG to 4.76 nm for the PUG-D) and also an appreciable increment of the invariant of scattering *Q* (from 0.022 for the PUG to 0.102 for the PUG-D) of the polymer. Therefore, the addition of the D as filler in the polyurethane foam induces a growth of the HS that participate to the polymer network, resulting in an improvement in the degree of the microphase separation of the PU.

### 4.2. Infrared Spectroscopy (FTIR)

**Figure 3:**
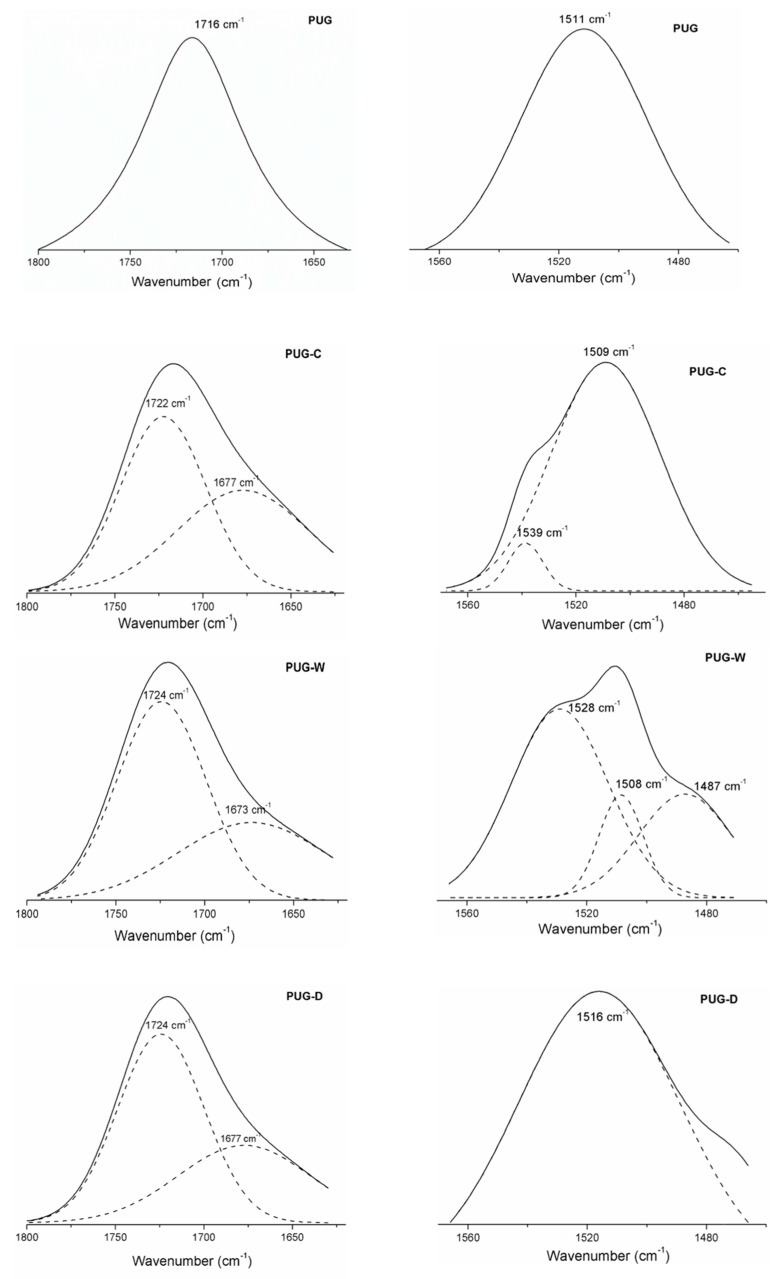
Deconvoluted spectra of produced foams.

The effect of filler addition on chemical structure of polyurethane foams was also evaluated by FTIR-ATR investigation. The main absorption vibration peaks of polyurethane composite foams (PUG-C, PUG-W and PUG-D) and pristine PUG are listed in Table 3. All the spectra highlight the disappearance of the characteristic NCO vibration peak (around 2270 cm^−1^) associated to the isocyanate consumption inherent to the urethanic linkage formation [10,11,30]. The characteristics band features of the mainly functional groups (urethanes and urea linkages), absorbed in the spectral region 1400–1800 cm^−1^, are analyzed and discussed and the main deconvoluted peaks are shown in Figure 3. For instance, as widely investigated [10,24,30,31] in the 1600–1800 cm^−1^ wavenumber region, the carbonyl vibration bands are correlated to the urethane and urea linkages, *H*-bonded (*H*-C=O) or free C=O (*f*-C=O) as summarized in the Table 3. Typically, *H*-C=O linkages are due to the HS domains present in the polyurethane structure formed between NH groups proton accepting (urethane or urea carbonyl), while the *f*-C=O linkages can exist dispersed in Soft segment (SS) or in interphase domains (urea phase).

In this region, the pristine PUG highlighted a stretching vibration peak related to *H*-C=O urethane linkages at 1716 cm^−1^; for the composite foams (PUG-C, PUG-W and PUG-D), the carbonyl region is split into two peaks. The *H*-C=O band of urethane disappears and the *f*-C=O correlated to urethane (around 1724–1722 cm^−1^) and urea (around 1677–1673 cm^−1^) linkages display (see the deconvoluted spectra in Figure 2) [31]. It was hypothesized that, the addition of fillers resulted in the decrease of the isocyanate (NCO) available to react with the polyol (to form HS) due to the reactions that can be occurred between the OH groups of fillers with NCO creating covalent-like bonds to the PU molecular chains. This leads to a decreasing of urethane density (reduction of *H*-C=O in urethane linkages) and to the formation of new urethane chains (*f*-C=O) and urea domains (*f*-C=O) more flexible [30,31,32].

Furthermore, the urethane domains for rigid polyurethane foams were also identified through the vibrational modes, *ν_as_* C-N and *δ* N-H, around 1511 cm^−1^ for PUG samples, at a lower wavenumber, 1509 cm^−1^, for the PUG-C and PUG-W. An increase in wavenumber for the PUG-D (1516 cm^−1^) was observed. This means that the addition of filler C and W induces a decreasing of networking in polyurethane structure, while the addition of D acts as cross-linking agent increasing the hardness of polyurethane chemical structure.

Apart from this, the spectra of PUG-C and PUG-W exhibit additional stretching vibration bands, around 1528–1539 cm^−1^, related to the NH of isolated urea domains (interphase) located in the polyurethane matrix that induce a reduction of the continuity of urethane structure.

### 4.3. Scanning Electron Microscopy (SEM)

**Figure 4:**
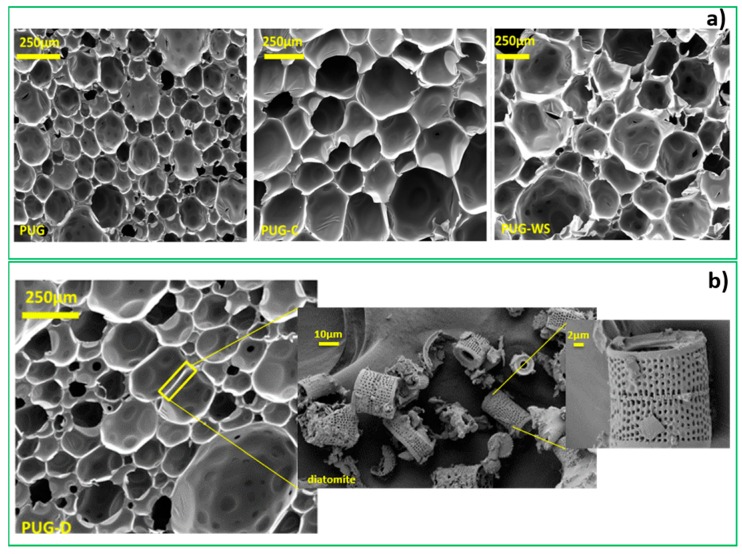
(**a**) SEM micrographs of PUG, PUG-C and PUG-WS. (**b**) SEM micrographs of PUG-D and the diatomite filler located within the cell-struts of PUG-D matrix.

The microstructure of the produced polyurethane foams (PUG, PUG-C, PUG-WS and PUG-D) was analyzed by SEM in order to determine the effect of several fillers on Polyurethane morphology. The SEM micrographs, showed in the Figure 4a,b, highlighted a homogeneous microstructure with a closed-cell porosity well distributed within the polyurethane matrix.

The cell diameters of PUG cross-section surface range from 160 µm to 250 µm, an increase of mean cell size was observed for the PUG-C and PUG-WS, for instance 200–350 µm values were recorded.

Conversely, for PUG-D (Figure 4b) a decrease in cell size (70–200 µm) was observed; indeed, for this composite material, the diatomite was able to induce a nucleation effect reducing the cell dimension of foam. It is also possible to identify within the cell-struts of the PUG-D foam matrix the presence of diatomite filler able to give a three-dimensional (3D) network entrapped in the cellular walls. The SEM micrographs of diatomite powder are shown in Figure 4a at high magnification. As can be observed, diatomite showed a very porous structure at nanometric scale ranging from 500–400 nm [20,33], typical sizes observed for aerogel structures. This typology of structure can considerably affect the thermal conductivity of the final composite foams [12].

### 4.4. Thermal Properties (Thermo-Gravimetric Analysis-TGA, Differential Scanning Calorimetric-DSC and Thermal Conductivity)

TGA analysis has been used as a tool to characterize the thermal degradation properties of polyurethane foams [29], it is important to point out that all the produced foams were manufactured without the addition of flame retardants. TGA results of produced samples are listed in Table 4. The thermograms of PUG and PUG composite foams show several degradation steps. The first degradation step occurs, for all polyurethane foam samples, in the range 150–220 °C (maximum of the derivative weight loss, Tmax1 around 220 °C) with a weight loss equal to 6wt% attributed to the elimination of bound water molecules. The second step which occurs in the temperature range of 250–350 °C (with Tmax2 equal to 311) is ascribed to the breaking of urethane links: the isocyanate dimerises to form carbodiimide with evolution of volatiles compounds as CO2, alcohols, amines etc. The carbodiimide is stable up to higher temperature when it degrades giving rise to the subsequently degradation steps (Tmax3).

As observed in Table 4, the maximum decomposition temperatures (Tmax) of the foams increased with the addition of filler. The enhancement of thermal stability for PUG-C and PUG-W can be ascribed to the high thermal stability of all fillers [12,34,35].

Furthermore, it is important to point out that for the PUG-WS and PUG-D a marked increase of the char (around 21 and 22.4 wt%, respectively) at high temperature (800 °C) with respect to the pristine PUG was detected. For the PUG-WS, this result could be ascribed to the presence of lignin in the walnut shell composition. For the PUG-D the further enhancement can be attributed to the presence of diatomite (inorganic) domains, which are able to reduce the amount of more-combustible organic molecules and to produce a siliceous layer that inhibits any mass transfer [12,34,35,36] producing more char component.

Finally, the higher amount of char, produced during the pyrolysis of composite foam, can potentially inhibit flame propagation through the substrate of polyurethane.

The thermal conductivity values for the produced foams range around 31 mW/mK, while for the sample PUG-D (diatomite) a reduction of the thermal conductivity, 27 mW/mK, with respect to the other foams, was recorded. This result is consistent with the ones reported in the literature for foam composites filled with preformed aerogel particles; in this case, the addition of diatomite reduces the thermal conductivity of the foams thanks to the intrinsic nanoporosity in its morphological structure. This behavior can be explained considering that the diatomite (located within the cell strut of the foam, see the micrographs in Figure 4b) due to its nanometric porosity (Figure 4b) includes voids into the solid phase of the cell-walls thus inducing a reduction of the radiative contribution λr of thermal conductivity. Generally, the thermal conductivity of a porous material can be described as a combination of different specific terms:

λ = λs + λg+ λr + λc [W/(m·K)]
(3)

where:

- λ is the apparent thermal conductivity of the insulation material,
- λs is the thermal conduction through the solid structure of the pore walls,
- λg is the conduction through the gas-filled inside the pores,
- λr is the heat radiation through the cell walls,
- λc is the convention within the cells, that is negligible.

The diatomite is able to reduce the λr parameter.

**Figure 5:**
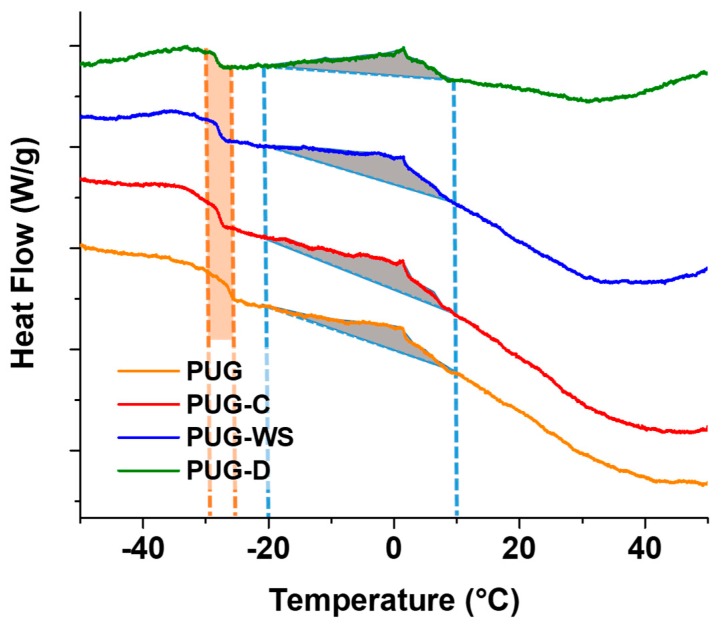
DSC thermograms of produced polyurethane foams.

DSC thermograms are reported in Figure 5 and the resulting data are summarized in Table 5. As observed in Figure 5, all the foams exhibit a glass transition temperature (T_g_) around 26 °C for the pristine PUG and a cold crystallization temperature (T_cc_) at 1.34 °C ascribed to the organized microphase (urea “nuclei”) present within the polyurethane foams.

By analyzing the DSC data of the composite foams, a slight decreasing in T_g_ and an increase of T_cc_ were recorded. Furthermore, an increase of the enthalpy values correlated to the cold crystallization phenomenon was mainly found for the PUG-C and PUG-W. These outcomes could be explained considering that cellulose, walnut shell (in particular) and diatomite both act as reactive fillers, subtracting isocyanate groups form the polyurethane reaction (because of the reaction of their OH groups with NCO), and thus, causing a decrease in the cross-linking and consequently of T_g_, and as nucleating agents leading to the increasing of T_cc_ and urea formation (ΔH). These confirm the SAXS/WAXS and FTIR analysis.

### 4.5. Mechanical Properties

**Figure 6:**
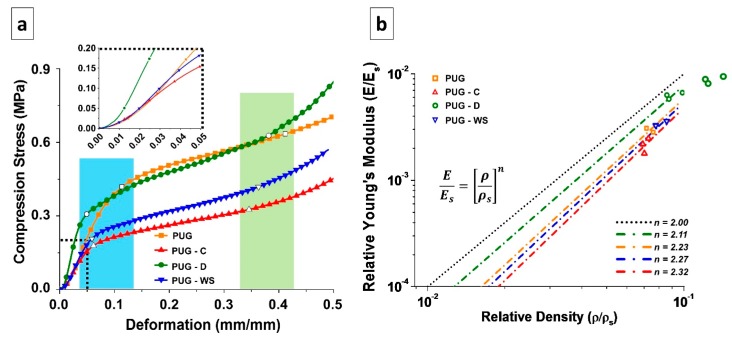
(**a**) Stress-strain curves and (**b**) graphical representation of Gibson-Ashby model of the foamed materials.

In order to evaluate the mechanical properties of PUG, PUG-C, PUG-WS and PUG-D polyurethane foams, compression tests were performed as well. The characteristic stress-strain curves of the analyzed materials are reported in Figure 6a. Each foam presents an elastomeric behavior, in which it is possible to appreciate three different regimes: linear elasticity (LE), elastic buckling (plateau, EB) and densification (DE) [37,38,39]. Depending on the adopted filler, those ranges present different limits. In the cyan area, the empty symbols on the curve represent the LE-EB transition point experienced by each material. On the other hand, in the light-green area the empty symbols are representative of the EB-DE transition points. The mechanical properties and the mechanical behavior of the polyurethane foam matrices are affected by the presence of cellulose, walnut shell and diatomite fillers, each in different ways, as detailed hereafter.

The LE part of the curve is mostly correlated to the intrinsic bending properties of the bulk material that constitutes the cell walls, which is initially deformed when a compression force is applied [37,38,39]. In the inset of the Figure 6a, it is possible to better appreciate the difference between the slopes of the initial linear part of each curve.

In the case of cellulose and walnut shell, the presence of the filler has a detrimental effect, in terms of elastic modulus and extent of the LE regime with respect to the pristine PUG foams. This can be ascribed to the chemical interaction of the aforementioned fillers with the PUG matrix, whose network results re-constructed, as also stated by SAXS-WAXS analysis. This kind of interaction has also induced the presence of cross-linked network with different crystalline domains, due to the presence of un-linked urea moieties (the urea is not bonded to the urethane polymer structure). Instead, when diatomite is added, the LE regime has the shortest length, while the elastic modulus results in the longest length. This is strictly related to this kind of filler, which is ceramic and rigid. Thanks to the presence of some OH groups (due to the few amount of Si-(OH) groups present in the chemical structure of diatomite), which could interact with the polyurethane structure (through isocyanate group) and alter the structure of the material (see the chemical characterization section), the D filler enhances the stiffness of the material constituting the cell walls, which is then well dispersed within the polymer matrix. This effect endows the bulk material with a completely new crystalline structure, involving the traditional polyurethanes cross-linking network and the different silica allotropic crystals of the diatomite, as stated by SAXS analysis.

The second part of the curve (EB) is characterized by the presence of a plateau. This behavior appears when the cell walls and edges of the foam, after being bent, start to distort, the foam cells appear to be twisted and the material loses its stiffness [37,38,39]. With respect to the PUG material, the others exhibit a transition point from LE regime to the EB one at lower stress and deformation values. From the EB portion of the stress-strain curve of the polyurethanes it is possible to evaluate the so-called Plateau Modulus, which gives information on the chemical structure of the material constituting the cell walls, and not on the foam density [37,38,39].

From the curves, it is possible to notice that the presence of the two strongly chemically interacting fillers (cellulose and walnut shells) produces materials with lower Plateau Modulus with respect to PUG (Table 6), with PUG-C having a lower slope than PUG-WS. In case of PUG-D, the extent of EB regime is quite similar to the neat material, further proving that the diatomite filler does not alter the chemical structure of the PUG, since it is not seizing much reactive sites of the isocyanate, as cellulose and walnut shells do instead. In this area, the stress at 10% deformation (σ10) is evaluated. As expected, PUG-C and PUG-WS has lower values, while PUG and PUG-D has practically the same value. These results are also in agreement with X-ray analyses, where different cross-linked networks were detected.

After the EB regime, the transition to DE occurs. In this last area, the slope of the curve starts to increase, and the stress escalates. Here, the opposing foam cells walls touch, being almost completely collapsed. If further strain compresses the material, the elastic modulus of the solid itself could be approached [37]. In this study, the strain stops at 50% of deformation, so that the DE regime is just at its beginning.

Even though the foam cell structure can be appreciated from the SEM analysis, the direct correlation between the cell walls or edges structure and mechanical properties is better elucidated by use of the Gibson-Ashby model [37,38]. For this model, relative young’s modulus and relative density must be considered. The first is given by the ratio between the elastic modulus of the foamed material (E) and the elastic modulus of the material constituting the cell walls (Es), while the second is the ratio between the foam density (ρ) and the density of the cell wall material (ρs), which was evaluated empirically, following the formula [40]:

(4) $$\frac{1}{\rho_{s}}=\frac{W_{m}}{\rho_{m}}+\frac{W_{f}}{\rho_{f}}$$

In Equation (4), *W_m_* and *ρ_m_* are the weight fraction and the density of the matrix, while *W_f_* and *ρ_f_* are the weight fraction and the density of the filler, respectively. In our case, the fillers’ densities (from data sheets) are *ρC* = 1500 kg/m^3^, *ρD* = 2300 kg/m^3^ and *ρWS* = 640 kg/m^3^, while the density of non-foamed PU is equal to 1200 kg/m^3^. The relationship between relative young’s modulus and relative density follows an exponential trend, as for the equation in Figure 6b, where the exponential n is a factor depending on the cell structure [39].

In Figure 6b, the Relative Young’s Modulus and Relative Density of the selected materials (empty symbols) are plotted. From these values, it is possible to obtain the n value at which the Gibson-Ashby model can be applied, and thus acquire information about the cell walls structure. For n = 2 this model is representative of the ideal behavior of the so-called open-cell polymer foam, in which only cell edges, and no cell walls, are present in the foam arrangement. Despite that most polyurethanes foams exhibit closed cells, their behavior follows the open-cell foams one, as in the cases studied here. This is related to the fact that, during manufacturing, the surface tension, which drives the formation of the cells foam, tends to let the material gather in the cell edges, leaving the cell walls thinned. Then, depending on the used filler, the behavior of each set of samples can be described by the Gibson-Ashby model, using different values of the exponent.

Therefore, in case of PUG, the exponent n results to be equal to 2.23, meaning that even if the material appears to have closed cells (Figure 4a), the thickness of the cell walls is lower than the one of the edges. In case of PUG-C and PUG-WS, the exponent n is slightly higher than that observed for PUG. This means that the material structure results more similar to the closed cells one, attributable to the distribution of the urea domains not only on the cell edges, but also on the cell walls. Despite that the Young’s Modulus of PUG-WS results were higher than the PUG one, the materials’ LE-EB transition point was actually lowered, as also pointed out by the σ-ε curves, since the urea domains should be located on the cell edges to contribute to the stiffness and to the strength of the material. However, in each case, the cell walls are so weak that they could be considered empty. For PUG-D, where the exponential n has the lowest value, the diatomite acts as a filler, whose high stiffness is exploited in the inclusion in the polymer matrix constituting the cell edges, which will then result as the real supporting column of the foam structure.

### 4.6. Comparison of Some Key Parameters of Selected Rigid Polyurethane Foams

**Figure 7:**
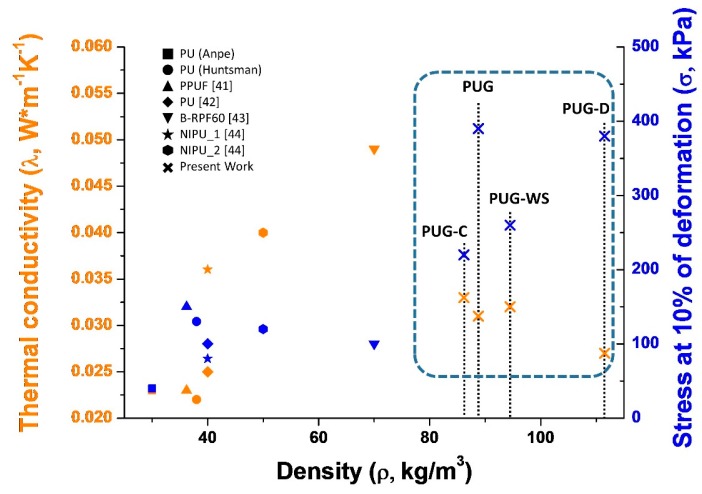
Comparison of density, thermal conductivity and mechanical properties of selected Polyurethane foams.

In Figure 7, a simple comparison of the key parameters that influence the overall impacts in the construction field (thermal conductivity, density and compressive strength evaluated at 10% of deformation) of composite PU foams produced in this study with those of selected conventional PUs (data from ANPE: *https://www.poliuretano.it/EN/index.html*, and technical data sheet from Huntsman: *https://www.huntsman.com/polyurethanes/a/Products*, PPUF [41] all foams were produced by using cyclopentane as blowing agent) and bio-based PUs (data from the recent literature: PU and B-RPF60 foams produced by replacing 40 wt% and 100 wt% of the synthetic polyol with bio-based polyol [42,43] respectively; NIPU_1 and NIPU_2 non-isocyanate foams [44]) is reported.

As observed in Figure 7, the replacement of a petrochemical precursors with renewable feedstock (mainly when the polyol is totally replacement, sample B-RPF60) in the synthesis of rigid PUR foams affect negatively the functional (increasing of λ for B-RPF60) and mechanical properties (the samples became brittle [41,42,43,44]) of produced foams with respect to the conventional PUs. Based on the literature, these outcomes could be correlated to the different cellular structure, of the porous material, induced by the complex chemical structure of the bio-based polyols.

Instead, in our case, the thermal conductivity of the PUG-D complies with that of commercial PUs, while the mechanical performance is superior, probably due to the higher density. For this reason they can, potentially, be used in the construction sector as thermal insulators.

## 5. Conclusions

Sustainable Sustainable Nanocomposite Polyurethane foams were successfully produced starting from Cardanol-based polyol. Walnut shell, cellulose and diatomite were used as natural nano-filler to tune chemico-physical, morphological and mechanical characteristics. The results show that with respect to the pristine polyurethane foam, walnut shell and cellulose, through their OH groups, react with isocyanate precursor producing a “polyurethane-like” chemical structure. This affects the thermal properties of the material with an improvement in thermal stability and the mechanical properties; in case of walnut shell, this causes an increase in stiffness similar to a flexible-like foam behavior.

Diatomite powder acts as reinforcing filler as it induces an increase in stiffness owing to the inclusion of filler in the polymer matrix constituting the cell edges, which will then result as the real supporting column of the foam structure. Furthermore, an improve in thermal stability with a prominent charring effect of the inorganic filler, and a reduction of thermal conductivity (around 13%) due to the decrease of cell-size microstructure and to presence of aerogel-like structure (diatomite) within the cell-struts of the foam, were also detected. These results prove the potentiality of these nanocomposite rigid foams as sustainable thermal insulators in construction applications.

## Figures and Tables

**Table 1:** PUG (Pristine polyurethane foam) and Composite foams formulations ([wt%]* with respect to 100 parts of total polyol. (PUG-C = Composite polyurethane with cellulose, PUG-WS = Composite polyurethane with walnut shell, PUG-D = Composite polyurethane with diatomite.).

Sample	Polyol [wt%]	MDI [wt%]	CH_3_COOK [wt%]	Niax L6164 [wt%]*	Niax PM40 [wt%]*	H_2_O [wt%]*	Powder [wt%]*	Amount of Bio-Based Components [wt%]
PUG	50	48	0.25	0.50	0.25	0.50	/	51.01
PUG-C	48	45	0.24	0.48	0.24	0.48	5	53.37
PUG-WS	48	45	0.24	0.48	0.24	0.48	5	53.37
PUG-D	48	45	0.24	0.48	0.24	0.48	5	53.37

**Table 2:** Values of the *q*_max_ (nm^−1^), *L* (nm), and *Q* for the polyurethane composite foams PUG, PUG-C, PUG-WS and PUG-D.

Sample	*q*_max_ (nm^−1^)	*L* (nm)	*Q*
PUG	1.42	4.42	0.022
PUG-C	1.18	5.32	0.018
PUG-WS	1.22	5.15	0.020
PUG-D	1.32	4.76	0.102

**Table 3:** Assignments of the main FT-IR bands of produced foams.

Assignment	Groups Assignment	Wavenumber [cm^−1^]			
		PUG	PUG-C	PUG-W	PUG-D
*υ_as_* C-N and *δ* N-H	Urethane linkages	1511	1509	1508	1516
*υ_as_* C-N and *δ* N-H	Urea linkages	-	1539	1528	-
Aromatic ring vibration	Urethane structure	1594	1594	1595	1593
*υ_s_* C=O	Urea bidentate	1611	1611	1611	1602
*υ_s_* C=O	*f*-C=O urea	-	1677	1673	1677
*υ_s_* C=O	*H*-C=O urethane	1716	-	-	-
*υ_s_* C=O	*f*-C=O urethane	-	1722	1724	1724

**Table 4:** Thermal properties form TGA and thermal conductivity of produced polyurethane foams.

Sample	Degradation Temperature [°C]				Weight Residues T [wt%]				λ [W/mK]
	T_max1_	T_max2_	T_max3_	T_char_	T_max1_	T_max2_	T_max3_	T_char_	
PUG	222	311	406	800	5.38	24.75	15.89	18.8	0.031
PUG-C	222	317	451	800	5.6	37.8	43.5	17.1	0.033
PUG-W	222	316	451	800	7	30	42	21	0.032
PU-D	270	310	440	800	13	24.6	40	22.4	0.027

**Table 5:** Glass Transition Temperatures and Enthalpy values at 0 °C of produced polyurethane foams.

Sample	Tg [°C]	Tcc [°C]	ΔH [J/g]
PUG	−26.25	1.34	1.364
PUG-C	−27.78	1.39	1.821
PUG-WS	−27.54	1.48	1.934
PUG-D	−28.35	1.53	1.614

**Table 6:** Mechanical and structural properties of the foams.

Sample	Young’s Modulus (E) [MPa]	Density (ρ) [kg/m^3^]	Cell Wall Density (ρ_s_) [kg/m^3^]	Stress at 10% deformation (σ_10_) [MPa]	Plateau Modulus (E_pl_) [MPa]
PUG	4.730 ± 0.29	88.75 ± 2.66	1200	0.39 ± 0.02	0.621 ± 0.028
PUG-C	3.431 ± 0.547	86.17 ± 2.08	1212	0.22 ± 0.04	0.446 ± 0.036
PUG-WS	5.501 ± 0.360	94.48 ± 4.69	1150	0.26 ± 0.04	0.587 ± 0.059
PUG-D	9.996 ± 0.690	111.53 ± 8.66	1229	0.38 ± 0.02	0.753 ± 0.004
